# Predictors of pharyngeal electrical stimulation treatment success in tracheotomised stroke patients with dysphagia: Secondary analysis from PHADER cohort study

**DOI:** 10.1016/j.neurot.2024.e00433

**Published:** 2024-08-23

**Authors:** Ivy Cheng, Philip M. Bath, Shaheen Hamdy, Paul Muhle, Satish Mistry, Rainer Dziewas, Sonja Suntrup-Krueger

**Affiliations:** a Academic Unit of Human Communication, Learning, and Development, Faculty of Education, The University of Hong Kong, Hong Kong; b Centre for Gastrointestinal Sciences, Faculty of Biology, Medicine and Health, University of Manchester, Manchester, UK; c Institute for Biomagnetism and Biosignal Analysis, University of Münster, Münster, Germany; d Stroke Trials Unit, Division of Clinical Neuroscience, University of Nottingham, Nottingham, UK; e Stroke, Nottingham University Hospital NHS Trust, Nottingham, UK; f Department for Clinical Research, Phagenesis Limited, Manchester, UK; g Department of Neurology, University Hospital Münster, Münster, Germany; h Department of Neurology, Klinikum Osnabrück GmbH, Osnabrück, Germany

**Keywords:** Dysphagia, Electrical stimulation, Pharynx, Stroke, Tracheotomy

## Abstract

Pharyngeal electrical stimulation (PES) has emerged as a promising intervention for neurogenic dysphagia, with potential benefits in reducing dysphagia severity in stroke patients. PES may facilitate decannulation in tracheotomised stroke patients with dysphagia, yet the predictive factors for treatment success have not been investigated in detail. This study used data from the PHAryngeal electrical stimulation for treatment of neurogenic Dysphagia European Registry (PHADER) study to identify predictive factors for PES treatment success among patients with post stroke dysphagia who required mechanical ventilation and tracheotomy. Multiple linear regression was performed to predict treatment success, as measured in improvement in dysphagia severity rating scale (DSRS), accounting for age, sex, stroke type, lesion location, baseline National Institutes of Health Stroke Scale (NIHSS) score, feeding status, time from stroke onset to PES, PES perceptual threshold and PES stimulation intensity at the first session. Cox regression was conducted to identify the predictors for decannulation for all participants. Ninety-eight participants (mean [SD] age ​= ​66.6 [13.0]; male 73.5%) were included in the analyses. Regression analyses showed that early intervention (*p* ​= ​0.004) and younger age (*p* ​= ​0.049) were significant predictors for treatment success. For participants who received PES during tracheotomy (n ​= ​60; mean [SD] age ​= ​66.6 [11.2]; male 73.3%), supratentorial stroke (*p* ​= ​0.033) and feeding status at baseline (*p* ​= ​0.025) were predictors of treatment success. Among all participants, early intervention was associated with higher likelihood of decannulation (*p* ​= ​0.026). These results highlight the importance of timely intervention, age and stroke location in PES treatment success for stroke patients with mechanical ventilation and tracheotomy.

## Introduction

Swallowing is a complex process mediated by multiple structures in the central nervous system (CNS), and damage to these structures due to neurological injury or neurodegenerative diseases can result in neurogenic dysphagia [1]. Dysphagia can lead to serious complications, including malnutrition, dehydration, aspiration pneumonia, prolonged hospital stays, poor psychosocial well-being, financial burden and death [[2], [3], [4]]. Stroke patients who are critically ill may receive endotracheal intubation and mechanical ventilation as a life-saving procedure. In the intensive care unit (ICU) environment, dysphagia is a significant risk factor for extubation failure, leading to potential reintubation, prolonged treatment, pneumonia and unfavourable outcomes in acute stroke patients [5]. Once tracheotomised, dysphagia is the most relevant obstacle to decannulation in these patients [6]. Moreover, following extubation, dysphagia (post-extubation dysphagia; PED) may develop, which further complicates the challenges experienced by critically ill and fragile ICU patients [7].

Pharyngeal electrical stimulation (PES) is a novel and innovative neurostimulation treatment for restoring the neurological control of swallowing in dysphagic patients that has been commercially available following Conformité Européenne (CE) certification in Europe in 2012 and more recently, was approved by the United States Food & Drug Administration (FDA) [8]. PES treatment involves electrically stimulating the pharyngeal mucosa through a catheter with two bi-polar electrodes that is passed through the nasal cavity to the pharynx [9]. Early physiological studies showed that a short (10 ​minutes) period of PES could induce persistent neuroplastic changes in the pharyngeal motor cortex, which is the key to functional recovery of swallowing following stroke [9,10]. Subsequent studies demonstrated the capacity of PES to modulate the swallowing system neurophysiologically [[11], [12], [13], [14]] and neurochemically [15]. Early phase II studies have shown that PES improved swallowing function, reduced aspiration, improved feeding status, and shortened hospital stays in patients with post stroke dysphagia (PSD) [11,13,16] and PED [17]. In patients with severe dysphagia who required tracheotomy, PES facilitates decannulation within 24–72 ​hours of treatment [18,19], which is associated with increase in saliva substance P [20]. By contrast, a phase III trial with subacute stroke patients reported neutral results, which may have been attributed to undertreatment and partial stimulation in the sham arm during the dose testing phase [21]. Nonetheless, a systematic review and meta-analysis suggested that PES showed a pooled overall beneficial effect in improving swallowing functions in patients with PSD [22].

Recently, the results of a large-scale multi-centre observational cohort study, the PHAryngeal electrical stimulation for treatment of neurogenic Dysphagia European Registry (PHADER), conducted across Austria, Germany and the United Kingdom were reported [23]. The results showed that PES significantly improved diet advancement by reducing dysphagia severity and the risk of penetration and aspiration in patients with neurogenic dysphagia. Nonetheless, the predictive factors for PES treatment success have not been explored in detail. Identifying these predictive factors for PES success is critical for clinical decision-making, providing clinicians with insights into which patients would benefit the most from PES treatment. We hypothesised that key baseline participant characteristics and treatment parameters would predict response to PES, especially in the subgroup of participants prior to decannulation. We performed a subgroup analysis of the PHADER population who required mechanical ventilation and tracheotomy.

## Materials and Methods

### The PHADER study

The current study analysed data collected from the PHADER study, a prospective single-arm observational clinical cohort study that took place between March 2015 and September 2018, in which all participants received PES [23]. The characteristics of the study population, outcome measures, primary statistical analyses, and main results for PHADER have been published previously [23]. A brief description of the PES intervention protocol, the primary outcome measure, and statistical analysis relevant to this study is given below.

### Participant characteristics

**Fig. 1:**
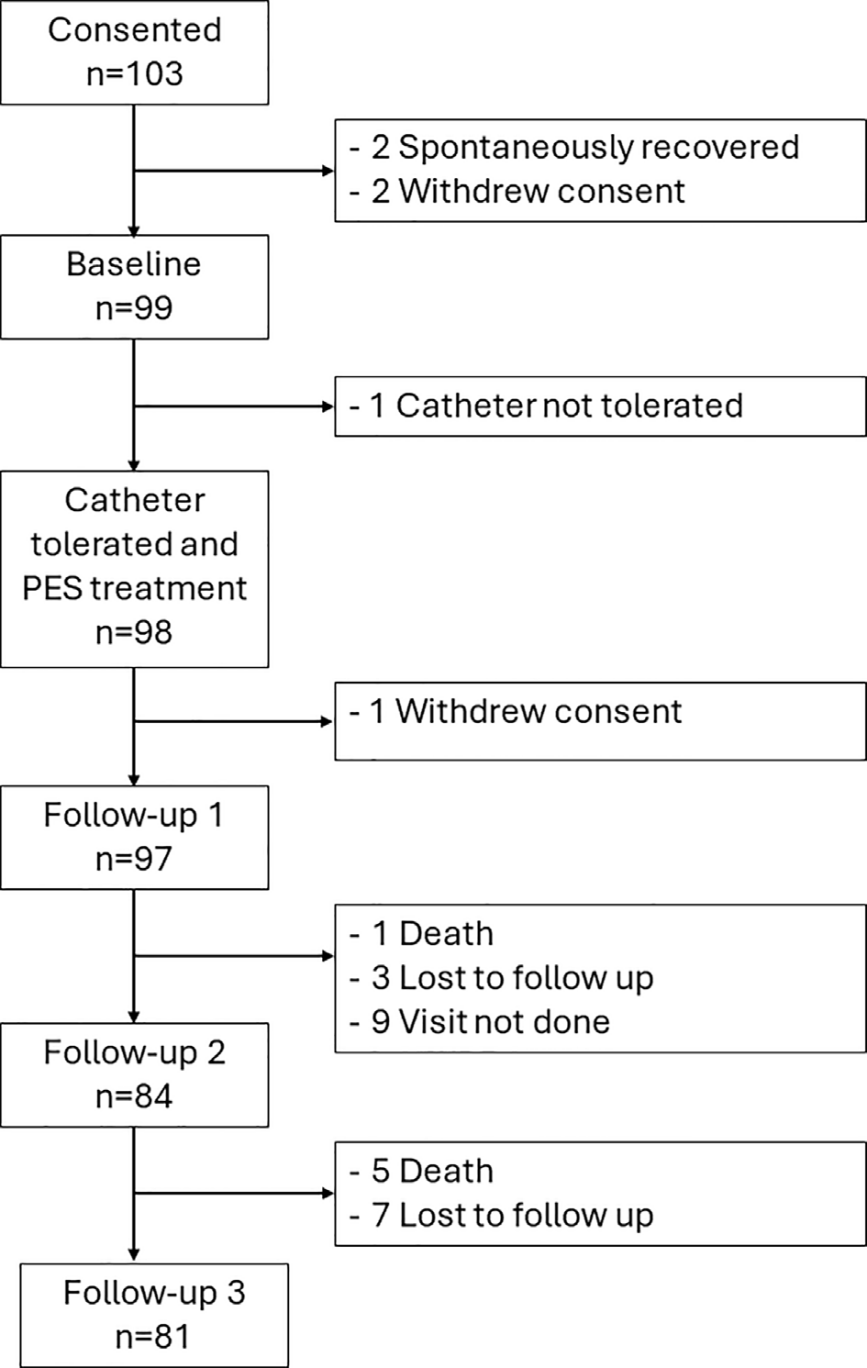
Total number of participants included in this subgroup analysis.

All participants recruited in the PHADER study had oropharyngeal dysphagia with a dysphagia severity rating scale (DSRS) [24] score of 6 or higher. Only participants with stroke who required mechanical ventilation and tracheotomy were included in this subgroup analysis. Initially, a total of 103 participants were recruited in the PHADER study (Fig. 1). After exclusion of spontaneous recovery and withdrawal of consent, ninety-nine participants underwent baseline assessments. One participant could not tolerate the PES catheter and was excluded for further analyses. Therefore, the data from 98 participants who received PES were included in this subgroup analysis.

### PES intervention protocol

PES was delivered using the Phagenyx® Neurostimulation System (Phagenesis Ltd., Manchester, United Kingdom). The treatment catheter is a specially designed single-patient use device with built-in stimulation electrodes and doubles as a feeding tube if necessary. Each participant received PES at 5 ​Hz at an intensity optimised by the Phagenyx® Neurostimulation System software and the operator for 10 ​minutes per day for three consecutive days [23]. The stimulation intensity was set at 75% of the maximal tolerable intensity above the perceptual threshold and was calibrated before commencement of PES on each treatment day.

### Primary outcome and assessment timepoints

The DSRS, which is a validated scale for swallowing impairment in patients with PSD, was used as the primary outcome measure [23,24]. Assessments were performed at baseline (on the day of screening; median [interquartile range; IQR] ​= ​1 [0–3] day before PES), and repeated at day 5, day 9, and 3 months (day 92) post-treatment. For patients with tracheotomy, decannulation followed the protocol used in the Pharyngeal electrical Stimulation for early decannulation in Tracheotomised stroke patients with neurogenic dysphagia (PHAST-TRAC) trial [19].

### Statistical analysis

All analyses were performed using IBM SPSS Statistics for Windows (Version 27.0). Multiple linear regression (MLR) was performed to predict treatment success at day 5, day 9 and 3 months (day 92) post-treatment, from (a) participant characteristics, including age, sex, stroke type (ischaemic versus haemorrhagic), lesion location (supratentorial versus infratentorial), baseline National Institutes of Health Stroke Scale (NIHSS) score and baseline feeding status (oral diet without supervision, oral diet under supervision, oral diet with support from staff, non-oral feeding with nasogastric tube (NGT) or nasojejunal tube (NJT), non-oral feeding with percutaneous endoscopic gastrostomy (PEG) tube or radiographically inserted gastrostomy (RIG) tube, or other feeding routes [25]); and (b) intervention characteristics, including time from stroke onset to PES, PES perceptual threshold and PES stimulation intensity at the first session. Treatment success was measured by changes in DSRS [24], from baseline to each assessment timepoint. MLR assumptions of linear relationship between outcome variables and independent variables, multivariate normality and absence of multicollinearity were tested, and these assumptions were not violated.

**Fig. 2:**
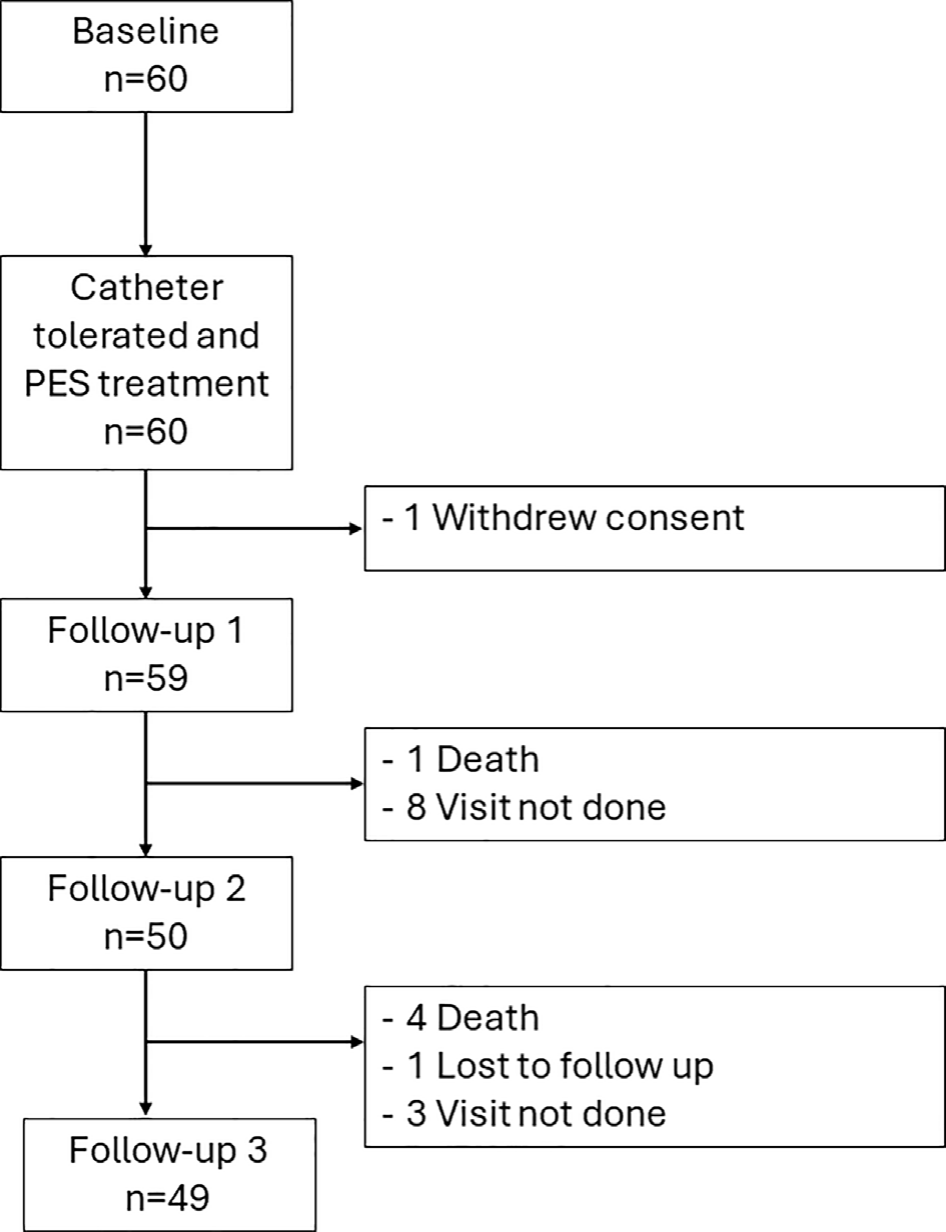
Number of participants who received pharyngeal electrical stimulation (PES) during tracheotomy.

Among the participants included in this subgroup analysis, some received PES during tracheotomy (n ​= ​60), while some received it during orotracheal intubation (n ​= ​18). The remaining (n ​= ​20) participants received orotracheal intubation or tracheotomy during hospital stay, but the orotracheal or tracheostomy tubes were removed prior to the first session of PES. A two-way mixed analysis of variance (ANOVA) was performed to analyse the interaction effects between the three subgroups and time. The effects of PES during orotracheal intubation have been reported in another study [26]. Therefore, further MLR analysis was performed with a focus on the subgroup of participants who received PES treatment only during tracheotomy (Fig. 2).

In addition to MLR, Cox regression was performed to analyse the relationship between the predictor variables, including age, sex, stroke type, lesion location, NIHSS at baseline, feeding status at baseline, PES perceptual threshold and stimulation intensity at the first session and the time from stroke onset to PES, and outcome, which is the odds of decannulation. Significance was set at *p* ​< ​0.05.

## Results

### Demographics

A total of 98 participants with stroke who required mechanical ventilation and tracheotomy (mean [standard deviation; SD] age ​= ​66.6 [13.0] years) were included in the analyses (Fig. 1). Most had an ischaemic stroke (75.5%), and the stroke lesion location was predominantly supratentorial (85.7%). All but one participant received alternative feeding at baseline. The average (SD) PES perceptual threshold and stimulation intensity on the first session were 15.9 (7.9) mA and 30.8 (10.2) mA respectively. Baseline characteristics, PES parameters and changes in DSRS for all participants included in this subgroup analysis are presented in Table 1.

**Table 1:** Baseline participant characteristics and intervention characteristics by group. Data are presented in mean (standard deviation), number (%) or median [interquartile range].

	All ventilated stroke patients (n ​= ​98)	Patients who received PES during tracheotomy (n ​= ​60)
Participant characteristics		
Age	66.6 (13.0)	66.6 (11.2)
Sex (Male / Female)	72 (73.5) / 26 (26.5)	44 (73.3) / 16 (26.7)
Body Mass index (BMI; kg/m^2^) by age groups		
50 years old or younger	24.6 (4.8)	25.8 (4.9)
51–60 years old	26.8 (9.1)	24.2 (4.6)
61–70 years old	26.2 (5.4)	26.1 (5.4)
71–80 years old	28.0 (4.4)	28.8 (3.6)
80 years old or older	26.5 (6.0)	26.4 (5.6)
Feeding status at baseline		
Oral, normal	0 (0.0)	0 (0.0)
Oral, supervision	1 (1.0)	0 (0.0)
Oral, with support	0 (0.0)	0 (0.0)
NGT or NJT	71 (72.4)	46 (76.7)
PEG or RIG	24 (24.5)	13 (21.7)
Other routes	2 (2.0)	1 (0.0)
NIHSS (/42)	13.4 (5.8)	13.5 (5.0)
Stroke type		
Ischaemic	74 (75.5)	46 (76.6)
Haemorrhagic	24 (24.5)	14 (23.3)
Stroke lesion location		
Supratentorial	84 (85.7)	54 (90.0)
Infratentorial	14 (14.3)	6 (10.0)
**Intervention characteristics**		
PES perceptual threshold at baseline (mA)	15.9 (7.9)	14.5 (7.6)
PES intensity on session 1 (mA)	30.9 (10.2)	30.0 (10.6)
Time from stoke onset to treatment (days)	29.5 [34.8]	32.5 [25.5]
**DSRS**		
Baseline	11.7 (1.2)	11.7 (1.2)
Day 5	10.8 (2.4)	11.1 (1.9)
Day 9	8.9 (3.8)	9.3 (3.6)
Day 92	5.3 (5.0)	5.6 (5.2)

DSRS: dysphagia severity rating scale [24]; NGT: nasogastric tube; NIHSS: National Institute Health Stroke Scale; NJT: nasojejunal tube; PEG: percutaneous endoscopic gastrostomy tube; PES: pharyngeal electrical stimulation; RIG: radiographically inserted gastrostomy tube.

### Predictors of treatment success among all participants with stroke who required mechanical ventilation and tracheotomy (n ​= ​98)

#### Participant characteristics

Results of MLR showed that age (*β* ​= ​0.118 [95%CI ​= ​0.000, 0.235]; *p* ​= ​0.049) was a significant predictor of treatment success at 3 months after PES (Table 2). This suggested that younger participants were more likely to have better treatment outcome at 3 months post-treatment than older participants. No other parameter was related to treatment outcome.

**Table 2:** Results of multiple linear regression analyses for all participants with stroke who required mechanical ventilation and tracheotomy.

	Change in DSRS at Day 5			Change in DSRS at Day 9			Change in DSRS at 3 months		
	*ß* [95% CI]	*SE*	*p*	*ß* [95% CI]	*SE*	*p*	*ß* [95% CI]	*SE*	*p*
**Participant characteristics**									
Age	-0.019 [-0.067, 0.030]	0.024	0.452	-0.016 [-0.096, 0.064]	0.04	0.691	0.118 [0.000, 0.235]	0.058	***0.049***∗
Sex	0.128 [-1.131, 1.387]	0.631	0.840	0.196 [-1.871, 2.264]	1.036	0.850	1.247 [-1.760, 4.253]	1.499	0.409
Type of stroke^a^	0.431 [-1.009, 1.871]	0.721	0.552	-0.147 [-2.512, 2.219]	1.185	0.902	1.596 [-1.929, 5.121]	1.757	0.368
Location of stroke lesion^b^	-0.043 [-1.656, 1.570]	0.808	0.958	-0.504 [-3.152, 2.145]	1.327	0.706	-3.306 [-7.670, 1.059]	2.176	0.135
NIHSS at baseline	-0.032 [-0.128, 0.065]	0.048	0.513	-0.060 [-0.219, 0.098]	0.079	0.451	-0.023 [-0.257, 0.210]	0.116	0.841
Feeding status at baseline^c^	0.391 [-0.576, 1.358]	0.485	0.422	0.271 [-1.318, 1.860]	0.796	0.735	2.118 [-0.159, 4.396]	1.135	0.068
**Intervention characteristics**									
PES perceptual threshold at baseline	-0.010 [-0.101, 0.080]	0.046	0.819	-0.029 [-0.177, 0.119]	0.075	0.697	-0.197 [-0.448, 0.054]	0.126	0.122
PES intensity at session 1	0.030 [-0.041, 0.100]	0.036	0.407	0.042 [-0.074, 0.157]	0.058	0.474	0.118 [-0.051, 0.287]	0.085	0.168
Time from onset to first PES	0.003 [-0.001, 0.007]	0.002	0.165	0.006 [-0.001, 0.014]	0.004	0.077	0.015 [0.005, 0.025]	0.005	***0.004***∗∗

CI: confidence interval; DSRS: dysphagia severity rating scale [24]; NIHSS: National Institute Health Stroke Scale; SE: standard error.

a 1 ​= ​ischaemic; 2 ​= ​haemorrhagic.

b 1 ​= ​infratentorial; 2 ​= ​supratentorial.

c 1 ​= ​Oral diet without supervision; 2 ​= ​Oral diet with supervision; 3 ​= ​Oral diet with support from staff; 4 ​= ​nasogastric tube or nasojejunal tube; 5 ​= ​percutaneous endoscopic gastrostomy tube or radiographically inserted gastrostomy tube; 6 ​= ​Other feeding routes.

f ∗ *p* < 0.05

g ∗∗ *p* < 0.01

#### Intervention characteristics

MLR showed that treatment characteristics collectively predicted treatment success at 3 months post-treatment (*F* (3, 72) ​= ​3.421, *p* ​= ​0.022, *R*^2^ ​= ​0.088). Among these variables, the time from stroke onset to treatment was the significant predictor in the model (*β* ​= ​0.015 [95%CI ​= ​0.005, 0.025]; *p* ​= ​0.004). This suggested that the earlier the participants received PES, the greater the chance for successful functional treatment outcome at 3 months was.

### Predictors for treatment success among participants who received PES during tracheotomy

#### Demographics

A subgroup of participants (n ​= ​60) who received PES during tracheotomy was identified for further analyses (Fig. 2). The mean (SD) age was 66.6 (11.2) years old (male: n ​= ​44 [73.3%]; female: n ​= ​16 [26.7%]). The majority of subgroup participants had ischaemic stroke (76.6%), and the stroke lesion location was predominantly supratentorial (90.0%). All subgroup participants received alternative feeding at baseline. The average (SD) PES perceptual threshold and stimulation intensity on the first session were 14.5 (7.6) mA and 30.0 (10.6) mA respectively. There were no significant interaction effects between time and those who received PES during tracheotomy and those who did not (*F* [4, 168] ​= ​0.921; *p* ​= ​0.453). The demographic characteristics are summarised in Table 1.

#### Participant characteristics

Table 3 presents the findings of the subgroup regression analyses. The results showed that age was a significant predictor of treatment success at Day 5 (*β* ​= ​0.048 [95%CI ​= ​0.006, 0.090]; *p* ​= ​0.025) and 3 months (*β* ​= ​0.235 [95%CI ​= ​0.086, 0.385]; *p* ​= ​0.003) after PES. This suggested that younger participants were more likely to have better treatment outcomes at Day 5 and at 3 months post-treatment than older participants. Furthermore, lesion location (*ß* ​= ​-8.065 [95%CI ​= ​-15.449, -0.681]; *p* ​= ​0.033) and baseline feeding status (*β* ​= ​3.706 [95%CI ​= ​0.504, 6.909]; *p* ​= ​0.025) were also significant predictor of treatment success at 3 months post-treatment. Participants with supratentorial stroke were more likely to have better treatment outcomes at 3 months after PES.

**Table 3:** Results of multiple linear regression analyses for a subgroup of participants who received pharyngeal electrical stimulation (PES) during tracheotomy.

	Change in DSRS at Day 5			Change in DSRS at Day 9			Change in DSRS at 3 months		
	*ß* [95% CI]	*SE*	*p*	*ß* [95% CI]	*SE*	*p*	*ß* [95% CI]	*SE*	*p*
**Participant characteristics**									
Age	0.048 [0.006, 0.090]	0.021	***0.025***∗	0.057 [-0.045, 0.159]	0.050	0.265	0.235 [0.086, 0.385]	0.073	***0.003***∗∗
Sex	0.099 [-0.889, 1.088]	0.489	0.840	-0.305 [-2.715, 2.106]	1.192	0.800	0.734 [-2.775, 4.244]	1.723	0.673
Type of stroke^a^	0.575 [-0.603, 1.753]	0.583	0.330	0.240 [-2.634, 3.113]	1.421	0.867	0.879 [-3.311, 5.070]	2.057	0.672
Location of stroke lesion^b^	0.166 [-1.337, 1.670]	0.743	0.824	-0.563 [-4.230, 3.104]	1.813	0.758	-8.065 [-15.449, -0.681]	3.625	***0.033***∗
NIHSS at baseline	-0.027 [-0.120, 0.067]	0.046	0.570	-0.012 [-0.241, 0.217]	0.113	0.917	-0.115 [-0.435, 0.204]	0.157	0.468
Feeding status at baseline^c^	0.581 [-0.356, 1.518]	0.463	0.217	-0.040 [-2.326, 2.246]	1.130	0.972	3.706 [0.504, 6.909]	1.572	***0.025***∗
**Intervention characteristics**									
PES perceptual threshold at baseline	-0.034 [-0.122, 0.055]	0.044	0.450	-0.073 [-0.260, 0.113]	0.093	0.433	-0.136 [-0.483, 0.211]	0.172	0.433
PES intensity at session 1	0.036 [-0.024, 0.096]	0.030	0.233	0.072 [-0.055, 0.199]	0.063	0.262	0.153 [-0.043, 0.350]	0.097	0.122
Time from onset to first PES	0.004 [-0.004, 0.011]	0.004	0.329	0.009 [-0.006, 0.024]	0.008	0.258	0.041 [0.007, 0.075]	0.017	***0.020***∗

CI: confidence interval; DSRS: dysphagia severity rating scale [24]; NIHSS: National Institute Health Stroke Scale; SE: standard error.

a 1 ​= ​ischaemic; 2 ​= ​haemorrhagic.

b 1 ​= ​infratentorial; 2 ​= ​supratentorial.

c 1 ​= ​Oral diet without supervision; 2 ​= ​Oral diet with supervision; 3 ​= ​Oral diet with support from staff; 4 ​= ​nasogastric tube or nasojejunal tube; 5 ​= ​percutaneous endoscopic gastrostomy tube or radiographically inserted gastrostomy tube; 6 ​= ​Other feeding routes.

d ∗ *p* < 0.05

e ∗∗ *p* < 0.01

Regarding feeding status, participants who received NJT or NGT feeding had better treatment outcomes compared to PEG tube or RIG tube feeding. Upon further analysis, participants who had NJT or NGT feeding received earlier treatment (mean days from stroke onset to treatment ​= ​30) than those who had PEG or RIG tube feeding (mean days from stroke onset to treatment ​= ​126; *p* ​< ​0.001). The feeding status also varied based on the protocols followed at various participating institutions.

#### Intervention characteristics

MLR revealed that the time from stroke onset to treatment was the significant predictor of treatment success (*β* ​= ​0.041 [95%CI ​= ​0.007, 0.075]; *p* ​= ​0.020) at 3 months after PES. This suggested that the earlier the participants received PES, the greater the chance for successful functional treatment outcome at 3 months was.

### Predictive factors for decannulation

**Fig. 3:**
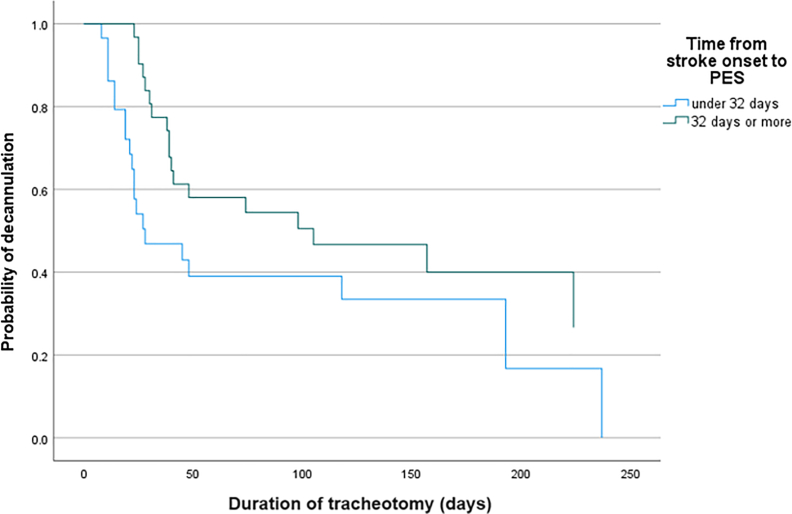
Graph showing cox regression results on the probability of decannulation with time from stroke onset to pharyngeal electrical stimulation (PES) as a predictive factor. The time from onset to treatment was stratified into under 32 days (median) and 32 days or above, represented by the separate lines.

Cox regression analysis showed that shorter time from stroke onset to PES was associated with a significantly higher odds of decannulation (Hazard Ratio ​= ​0.982 [95% CI ​= ​0.966, 0.998]; *p* ​= ​0.026) (Fig. 3). Other variables showed no significant results.

## Discussion

In this study, we performed regression analyses on the data collected from the PHADER study to evaluate predictive factors for PES treatment success in dysphagic stroke patients who required ventilation and tracheotomy. We found that early treatment and young age were predictors for treatment success with respect to improvement in swallowing function. In addition, supratentorial stroke, and NJT or NGT feeding rather than RIG or PEG tube feeding at baseline, were predictive of treatment success in a subgroup of participants who received PES during tracheotomy. Regarding the chance of decannulation, early treatment is the single factor associated with higher odds of decannulation. These findings provided significant insights into the therapeutic value and clinical application of PES.

### Early dysphagia intervention and neuroplasticity following stroke

Shorter time from stroke onset to PES treatment has consistently been identified as a predictive factor for treatment success across analyses. This finding is supported by previous studies which described the neuroplasticity mechanisms that occur in the CNS following stroke. Neurological and functional recovery after stroke typically takes place in the first three months, with most rapid recovery occurring within the first two weeks [27]. During this period, several stages of repair processes from cellular to brain network levels take place that eventually drive functional recovery [28]. In the context of dysphagia, Hamdy and colleagues [10,29] found that functional recovery of swallowing occurred spontaneously within the first three months of stroke onset in patients with PSD, and such recovery was associated with increase in pharyngeal cortical representation in the unaffected hemisphere, suggesting the importance of neuroplasticity in recovery. Given that recovery takes place most rapidly during the first three months post stroke, the timing of dysphagia intervention therefore plays an important role in functional outcomes in patients with PSD. Studies have shown that early (within 3 days of stroke) swallowing therapy leads to better functional outcome, reduced chest infection and better survival than late intervention in patients with PSD [30,31]. Regarding the effects of PES, a recent systematic review and meta-analysis revealed that the treatment effects of neurostimulation treatments were most significant when applied during the first two weeks of stroke [22]. Taken together, application of interventions that induce neuroplastic changes, such as PES, during the early phase of post-stroke recovery may promote the natural neurological recovery processes, leading to better treatment outcomes. Therefore, early identification of dysphagia and initiation of treatment is recommended in clinical practice. Our finding mirrors that found across PHADER as a whole [23] where earlier treatment was associated with a greater reduction in DSRS.

### Age

We found that young age is a predictive factor for better treatment outcomes. Presbyphagia, which refers to age-related changes in swallowing, may play a role in the observed findings in our study cohort [32]. Alterations in swallowing physiology due to increasing age, for example reduced gustatory, olfactory and pharyngeal sensation, and diminished skeletal muscle mass and quality, is termed primary presbyphagia and is considered non-pathological [32]. By contrast, disordered swallowing that occurs in the elderly people in the presence of other comorbidities such as stroke is referred to as secondary presbyphagia [33]. In acute stroke patients, a recent study showed that the volumes of muscles involved in swallowing decreased with increasing age and were inversely related to dysphagia severity, suggesting that PSD may be complicated by age-related atrophy of swallowing muscles in older patients [34]. Furthermore, studies have found that the cortical neuroplasticity mechanisms became less efficient with ageing, which may potentially lead to less responsiveness towards neuromodulatory treatments such as PES [35]. Therefore, it is possible that the treatment outcomes for the older patients in our cohort may be affected by secondary presbyphagia and reduced efficiency in neuroplastic mechanisms, such that they are less likely to benefit from PES than the younger patients. However, age was not found to be associated with lower DSRS across PHADER as a whole [23] so our finding may reflect chance or be a finding specific to ventilated stroke patients. Nonetheless, following the results for the PHAST-TRAC trial, which was a randomised controlled trial that found that PES could facilitate decannulation in ventilated stroke patients, the device labelling of the Phagenyx® Neurostimulation System was changed. The system now allows more PES treatments (up to six 10-minute sessions) to be delivered compared to when PHADER was conducted (three 10-minute sessions). The extra treatment sessions might offer these older or more treatment-resistant patients additional opportunities to generate beneficial neuroplasticity.

### Supratentorial stroke

Among the patients who received PES during tracheotomy, supratentorial stroke was a predictive factor of treatment success. This may imply that patients who have less severe dysphagia may have better treatment outcome. While both supratentorial and infratentorial structures are essential for the neural control of swallowing and damage to these structures can lead to dysphagia [1], some studies suggested that infratentorial (predominantly brainstem) lesions are associated with pharyngeal phase dysfunction and higher occurrence of penetration and aspiration than supratentorial lesions [1]. This is not surprising given that the brainstem is where the central pattern generator for swallowing, which mediates the swallowing response pattern, is located. Moreover, it is plausible that although spontaneous and PES-driven neuroplasticity changes may occur at the cortical level, such reorganisation may not result in effective improvement in swallowing if the brainstem, where all descending fibres project to, is damaged in patients with infratentorial stroke. Therefore, in our study cohort, patients with supratentorial lesions may have relatively less severe dysphagia and responded better to PES compared to those with infratentorial stroke. However, supratentorial stroke was not found to be associated with lower DSRS across PHADER as a whole [23] so our finding may reflect chance or be a finding specific to ventilated stroke patients.

### Study strengths and limitations

The strengths of this study are the large sample size from a multi-centre, international clinical study, and the consistent findings across research centres, suggesting high external and internal validity of the findings. Moreover, this study focussed on the data of stroke patients who required tracheotomy, which allowed a better understanding of the effects of PES for this population and the optimal factors that facilitate treatment success. Nonetheless, this study is limited by the retrospective nature of the analyses in which potential confounding variables cannot be fully elucidated. Information such as comorbidities and changes in medical conditions other than dysphagia was not collected in the study. The PHADER study in which this subgroup analysis is based on was limited by the lack of a control group for comparison of treatment effects. The observed improvement may be partly due to natural recovery [23]. However, given that the treatment typically started several weeks after stroke onset in this subgroup where dysphagia was relatively stable at baseline, the rapid improvement in DSRS following PES suggested that the improvement cannot be solely attributed to natural recovery.

In conclusion, we performed a secondary analysis using the data from the PHADER study to identify predictive factors for PES treatment success in stroke patients who required mechanical ventilation and tracheotomy. Our findings revealed that early intervention and younger age were key predictors for treatment success. Moreover, for a subgroup of participants who underwent PES during tracheotomy, supratentorial stroke and feeding status at baseline were found to be significant indicators of treatment success. Importantly, early intervention was strongly associated with higher odds of decannulation. These results provided valuable insights into the therapeutic efficacy and practical implications of using PES as a treatment for stroke patients with mechanical ventilation, highlighting the benefits of timely intervention and individualised treatment planning.

## Ethics approval and consent to participate

Not applicable.

## Consent for publication

Not applicable.

## Funding

This study did not receive any funding from agencies in the public, commercial, or not-for-profit sectors.

## Data availability

Not applicable.

## Author contributions

Dr Bath had full access to all of the data in the PHADER study and takes responsibility for the integrity of the data and the accuracy of the data analysis across the whole study but not this subgroup analysis.

Concept and design: Cheng, Bath, Dziewas, Hamdy, Mistry, Suntrup-Krueger.

Analysis and interpretation of data: Cheng, Bath, Dziewas, Hamdy, Muhle, Mistry, Suntrup-Krueger.

Drafting of the manuscript: Cheng, Suntrup-Krueger, Dziewas.

Critical revision of the manuscript for important intellectual content: Cheng, Bath, Dziewas, Hamdy, Muhle, Mistry, Suntrup-Krueger.

Supervision: Bath, Hamdy, Dziewas, Suntrup-Krueger.

## Availability of data and materials

Not applicable.

## Declaration of competing interest

The authors declare the following financial interests/personal relationships which may be considered as potential competing interests: Professor Philip M Bath reports a relationship with British Heart Foundation that includes: funding grants. Professor Philip M Bath reports a relationship with UK Research and Innovation Medical Research Council that includes: funding grants. Professor Philip M Bath reports a relationship with Phagenesis Ltd that includes: consulting or advisory. Professor Philip M Bath reports a relationship with Diamedica OÜ that includes: consulting or advisory. Professor Philip M Bath reports a relationship with Moleac Pte Ltd that includes: consulting or advisory. Professor Philip M Bath reports a relationship with Sanofi that includes: consulting or advisory. Professor Philip M Bath reports a relationship with Nestle HealthCare Nutrition Inc that includes: consulting or advisory. Professor Shaheen Hamdy reports a relationship with Phagenesis Ltd that includes: board membership and equity or stocks. Professor Shaheen Hamdy reports a relationship with UK Research and Innovation Medical Research Council that includes: funding grants. Professor Shaheen Hamdy reports a relationship with National Institute for Health and Care Research that includes: funding grants. Professor Shaheen Hamdy reports a relationship with Wellcome Trust that includes: funding grants. Professor Shaheen Hamdy reports a relationship with National Institute for Health and Care Excellence that includes: board membership and non-financial support. Dr Satish Mistry reports a relationship with Phagenesis Ltd that includes: employment. Professor Dr. Rainer Dziewas reports a relationship with Bayer AG that includes: consulting or advisory. Professor Dr. Rainer Dziewas reports a relationship with Boehringer Ingelheim GmbH that includes: consulting or advisory. Professor Dr. Rainer Dziewas reports a relationship with Daiichi Sankyo Inc that includes: consulting or advisory. Professor Dr. Rainer Dziewas reports a relationship with Nestle HealthCare Nutrition Inc that includes: consulting or advisory. Professor Dr. Rainer Dziewas reports a relationship with Olympus Corporation that includes: consulting or advisory. Professor Dr. Rainer Dziewas reports a relationship with Sanofi that includes: consulting or advisory. Professor Dr. Rainer Dziewas reports a relationship with Pfizer Inc that includes: consulting or advisory. Univ.-Prof. Dr. med. Sonja Suntrup-Krueger reports a relationship with German Research Foundation that includes: funding grants. Univ.-Prof. Dr. med. Sonja Suntrup-Krueger reports a relationship with Else Kroner-Fresenius Foundation that includes: funding grants. If there are other authors, they declare that they have no known competing financial interests or personal relationships that could have appeared to influence the work reported in this paper.

Dr Bath is Stroke Association Professor of Stroke Medicine and is a National Institute for Health Research (NIHR) Senior Investigator; he reports receiving grant funding from the British Heart Foundation and Medical Research Council (MRC), was a co-Chief Investigator of PHADER and reports personal fees from Phagenesis, DiaMedica, Moleac, Sanofi and Nestle.

Dr Hamdy is Chief Scientific Officer of Phagenesis Ltd; he is a board director, holds shares in Phagenesis Ltd; he reports receiving grant funding from the MRC, NIHR and Wellcome Trust; he was also a NICE MTAC committee member and interim chair until October 2022 and reviews medical technologies for potential guidance for use in the NHS, UK.

Dr Mistry is an employee of Phagenesis Ltd.

Dr Dziewas was a co-Chief Investigator of PHADER and reports receiving honoraria/fees from Bayer, Boehringer Ingelheim, Daiichi Sankyo, Nestle, Olympus, Sanofi and Pfizer.

Dr Suntrup-Krueger is supported with an endowed professorship by the Else Kröner-Fresenius-Stiftung and reports receiving grants from the German Research Foundation (DFG).

The remaining authors have no declarations.
